# Characteristics and Health Care Utilization of Patients With Housing Insecurity in the ED

**DOI:** 10.1001/jamanetworkopen.2024.8565

**Published:** 2024-04-26

**Authors:** Madeleine A. Z. Ball, Daniel E. Sack, Sophia A. Druffner, Ian Jones, Jesse O. Wrenn, Mitchell M. Sexton, Marybeth Shinn, Jennifer J. Hess

**Affiliations:** 1 Vanderbilt University School of Medicine, Nashville, Tennessee; 2 Department of Medicine, University of Pennsylvania, Philadelphia; 3 Vanderbilt University Peabody College, Nashville, Tennessee; 4 Department of Emergency Medicine and Biomedical Informatics, Vanderbilt University Medical Center, Nashville, Tennessee; 5 Department of Emergency Medicine, Vanderbilt University Medical Center, Nashville, Tennessee

## Abstract

**Importance:**

Unstable housing and homelessness can exacerbate adverse health outcomes leading to increased risk of chronic disease, injury, and disability. However, emergency departments (EDs) have no universal method to identify those at risk of or currently experiencing homelessness.

**Objective:**

To describe the extent of housing insecurity among patients who seek care in an urban ED, including chief concerns, demographics, and patterns of health care utilization.

**Design, Setting, and Participants:**

This cross-sectional study included all adult patients presenting to the ED at Vanderbilt University Medical Center (VUMC), an urban tertiary care, level I trauma center in the Southeast US, from January 5 to May 16, 2023.

**Main Outcomes and Measures:**

The primary outcome was the proportion of ED visits at which patients screened positive for housing insecurity. Secondary outcomes included prevalence of insecurity by chief concerns, demographics, and patterns of health care utilization.

**Results:**

Of all 23 795 VUMC ED visits with screenings for housing insecurity (12 465 visits among women [52%]; median age, 47 years [IQR, 32-48 years]), in 1185 (5%), patients screened positive for current homelessness or housing insecurity (660 unique patients); at 22 610 visits (95%), the screening result was negative. Of visits with positive results, the median age of patients was 46 years (IQR, 36-55 years) and 829 (70%) were among male patients. Suicide and intoxication were more common chief concerns among visits at which patients screened positive (132 [11%] and 118 [10%], respectively) than among those at which patients screened negative (220 [1%] and 335 [2%], respectively). Visits with positive results were more likely to be among patients who were uninsured (395 [33%] vs 2272 [10%]) and had multiple visits during the study period. A higher proportion of positive screening results occurred between 8 pm and 6 am. The social work team assessed patients at 919 visits (78%) with positive screening results.

**Conclusions and Relevance:**

In this cross-sectional study of 23 795 ED visits, at 5% of visits, patients screened positive for housing insecurity and were more likely to present with a chief concern of suicide, to be uninsured, and to have multiple visits during the study period. This analysis provides a call for other institutions to introduce screening and create tailored care plans for patients experiencing housing insecurity to achieve equitable health care.

## Key Points

**Question:**

What is the scope of housing insecurity for patients presenting to a major emergency department in the Southeast US?

**Findings:**

In this cross-sectional study of 23 795 emergency department visits, in 5% of visits, patients screened positive for housing insecurity. Patients experiencing homelessness were more likely to present with a chief concern of suicide, to be uninsured, and to have multiple visits during the study period.

**Meaning:**

This study supports screening for homelessness in emergency departments, directing resources toward mental health care, and focusing on high utilization as vital steps in optimizing care for patients with housing insecurity.

## Teaser

This cross-sectional study describes the proportion of emergency department (ED) visits at which patients tested positive for housing insecurity.

## Introduction

More than 653 000 people experienced homelessness in the US on a single night in 2023,^1^ and more than 1.2 million people in the US spent at least 1 night in an emergency shelter or transitional housing program in 2021.^2^ Homelessness in Tennessee increased by 45.6% from 2020 to 2022, the third largest increase among states.^1^ On a single night in January 2023, 2129 people were identified as homeless in Nashville via a point-in-time estimate.^3^ Given the difficulty of counting this population, these numbers likely underestimate the true population of individuals experiencing homelessness.^4^

Unstable housing and homelessness can exacerbate adverse and inequitable health outcomes. Individuals experiencing homelessness are at greater risk of chronic diseases, infectious diseases, injury, and disability,^5,6,7,8^ leading to increased acute hospital visits and emergency department (ED) morbidity and mortality.^9,10^ Even as recognition of housing as a social determinant of health expands,^11,12,13,14^ housing status is infrequently addressed when creating care plans.^15^ Reliable, accurate, and timely identification of those at risk of or currently experiencing homelessness is vital for effective interventions and safe discharge.

Paradoxically, there is no universal method in health care settings to identify patients experiencing homelessness despite regulatory movement to increase hospital-level screening for social determinants of health.^16^ In 2012, the Veterans Health Administration began to use the Homelessness Screening Clinical Reminder (HSCR), a 2-question screening tool, within its outpatient clinics. These questions asked:

1. In the past 2 months, have you been living in stable housing that you own, rent, or stay in as part of a household? (Negative response indicates current unhoused status.)
2. Are you worried or concerned that in the next 2 months you may not have stable housing that you own, rent, or stay in as part of a household? (Positive response indicates risk of future homelessness.)

This screening was implemented and validated in the Veterans Health Administration system, where question 2 predicted future shelter entry.^17^ Following the HSCR implementation, clinicians reported that this information influenced clinical decision-making and care plans.^18^ In 2023, the Centers for Medicare & Medicaid Services introduced mandatory screening for social determinants of health, including housing instability, for admitted patients but not for EDs.^16^

Although screening tools are being implemented,^19,20^ to our knowledge, few studies have described patients at risk of or currently experiencing homelessness who present to an urban ED using a systematic screening method. Even fewer have assessed prevalence of homelessness among individuals presenting to acute care centers. Those that have estimated prevalence reported that 0.7% to 35% of patients presenting to multiple care settings experience homelessness.^4,21,22,23^

This study quantified the prevalence of homelessness and housing insecurity among all patients who sought care in an urban ED in the Southeast US using the HSCR. We describe the demographics, method of arrival, diagnoses, acuity, timing of presentation, disposition, and insurance status for ED patients experiencing homelessness. In addition, given that about one-fourth of people experiencing homelessness in Nashville are unsheltered,^3^ we examined whether weather was associated with ED visits. Finally, because patients experiencing homelessness use EDs more frequently than others,^15,24,25^ we examined repeat visits. These data may provide a baseline to guide future hypothesis-driven interventions.

## Methods

This cross-sectional study was performed at the Vanderbilt University Medical Center (VUMC), an academic tertiary care center in Nashville, Tennessee. The adult ED serves as a level I trauma center and a referral center for people in Tennessee and adjoining states.^26^ Before this study, there was no systematic ED screening for housing insecurity. After institutional approval, we added the HSCR questions to existing ED registration and triage processes. The VUMC institutional review board granted an exemption for this study, including patient consent, as the data were collected as part of routine clinical care. This study followed the Strengthening the Reporting of Observational Studies in Epidemiology (STROBE) reporting guideline for cross-sectional studies.^27^

The HSCR screening was administered by registration staff during ED visits. All patients who answered “no” to question 1 were coded as homeless. Remaining patients who answered “yes” to question 2 were coded as unstably housed. Either designation led to an automatic notification of the ED social work team. For analysis, we grouped those who screened positive for current homelessness and risk of future homelessness into 1 category coded as insecure housing (Table 1).

**Table 1.** Coding of Responses to Homelessness Screening Clinical Reminder

Question 1: In the past 2 mo, have you been living in stable housing that you own, rent, or stay in as part of a household?	Question 2: Are you worried or concerned that in the next 2 mo you may not have stable housing that you own, rent, or stay in as part of a household?	Interpretation	Result
Yes	No	Stably housed	Secure housing (negative screening result)
Yes	Yes	Unstably housed	Insecure housing (positive screening result)
No	Yes or no	Homeless	

In this descriptive, cross-sectional study, data were collected on VUMC ED adult patients (aged ≥18 years) for visits from January 5, 2023, when the screening was implemented, to May 16, 2023, when procedures changed (from registration staff screening to patient self-report). Additionally, we collected data directly from the electronic medical record (EMR) on patient age, race (self-reported using EMR-coded categories during patient registration), ethnicity (self-reported), gender (self-reported), preferred language, insurance status, ED visit date, arrival time and method, presenting symptom, primary discharge diagnosis, disposition plan, and whether a member of the social work team was able to see each patient who screened positive on either question. Race categories included American Indian or Alaska Native; Asian; Black or African American; Hispanic, Latino, or Latina (we relied on categories in the EMR for the race categories, and some people identified their race as Hispanic or Latino while others may have defined their race as White and their ethnicity as Hispanic or Latino; there are different numbers as some patients left fields blank while others did not); Middle Eastern or North African; White; multiracial; other (those with response of “none of these,” “unable to provide,” and “prefer not to answer”); and unknown. Ethnicity categories were Hispanic, Latino, or Latina; not Hispanic, Latino, or Latina; other (those with response of “none of these,” “unable to provide,” and “prefer not to answer”); and unknown. Race and ethnicity were included given that historical marginalization of racial and ethnic minority populations puts these individuals at greater risk. We recognize that race and ethnicity are social constructs; however, structural racism is linked to medical outcomes in the US. This phenomenon is particularly salient for individuals experiencing homelessness. In our article, we are not implying biologic significance, but include race to bring attention to racial and ethnic disparities present in this patient population. Gender categories included female, male, and unknown. The Emergency Severity Index (ESI) triage score, a standardized method of assessing acuity that ranges from ESI level 1 (most critically ill) to level 5 (least critically ill),^28^ was collected at admission. We categorized individual chief concerns and primary ED diagnoses for each visit. Finally, we downloaded Nashville International Airport weather station data from the National Oceanic and Atmospheric Administration.^29^

### Statistical Analysis

We first present descriptive statistics, stratified by housing status (secure housing, insecure housing, and missing) for patient characteristics by visit. We then assessed how many unique patients visited the VUMC ED during the study period and how many of the encounters were for patients with 1, 2, 3, 4, or 5 or more visits. We repeated the descriptive analysis for patients who presented more than 3 times during the 4.5-month study period (defined as high utilization). We then aggregated housing screening data within each month, day of the week, and time of day to describe the prevalence and timing of visits. We generated prevalence 95% CIs from 1000 bootstrapped samples. We used locally estimated scatterplot smoothing to assess the association between daily prevalence by daily minimum, maximum, and average temperature and precipitation.

Data were analyzed in R, version 4.2.2 (R Project for Statistical Computing).^30^ The R code is available for review online.^31^

## Results

From January 4 to May 16, 2023, a total of 23 795 VUMC ED housing screenings were completed for adult patients as a part of standardized patient registration (12 465 visits among females [52%], 11 329 [48%] among males, and 1 [<1%] among those with unknown gender]; median age, 47 years [IQR, 32-48 years]). Of the total visits, less than 1% were among American Indian or Alaska Native patients; 2% among Asian patients; 22% among Black or African American patients; 5% among Hispanic, Latino, or Latina patients; less than 1% among Middle Eastern or North African patients; 68% among White patients; 1% among multiracial patients; and less than 1% among patients with other race. By ethnicity, 8% of visits were among Hispanic, Latino, or Latina patients; 91% among non-Hispanic, non-Latino, or non-Latina patients; and less than 1% among those with other ethnicity. Of 1185 visits with positive screening results (5% [660 unique patients]), at 1065, patients screened positive for homelessness, and at 120, patients screened positive for unstable housing. An additional 2776 visits (10%) were missing a screening secondary to severe trauma, patients leaving without being seen, or discharge prior to screening completion.

Of visits with positive screening results, the median age of patients was 46 years (IQR, 36-55 years); 356 (30%) were among female patients and 829 (70%) among male patients. At 22 610 visits (95%), the screening result was negative. Positive screening results were more likely among visits for people aged 35 to 64 years (853 [72%]) compared with screening negative for homelessness and/or housing instability (10 400 [46%]) and less likely among those aged 75 years or older (6 [0.5%] vs 2436 [11%]) (Table 2).^32,33^ They were also more likely among visits by male patients (829 [70%] vs 10 500 [46%]), Black patients (372 [31%] vs 5035 [22%]), and those insured by Medicaid (326 [28%] vs 3081 [14%]) or uninsured (395 [33%] vs 2272 [10%]). Demographic data are presented graphically in eFigure 1 in Supplement 1.

**Table 2.** Characteristics of Patients With Visits to the ED at Vanderbilt University Medical Center Between January 5 and May 16, 2023, by Housing Status

Characteristic	Visits, No. (%)		
	Secure housing (n = 22 610)	Insecure housing (n = 1185)	Missing (n = 2776)
Age, median (IQR), y	48 (32-64)	46 (36-55)	41 (29-59)
18-24	2697 (12)	45 (3.8)	402 (14)
25-34	3974 (18)	220 (19)	673 (24)
35-44	3610 (16)	285 (24)	491 (18)
45-54	3256 (14)	333 (28)	366 (13)
55-64	3534 (16)	235 (20)	360 (13)
65-74	3103 (14)	61 (5)	227 (8)
≥75	2436 (11)	6 (1)	257 (9)
Gender			
Female	12 109 (54)	356 (30)	1310 (47)
Male	10 500 (46)	829 (70)	1400 (50)
Unknown	1 (<0.1)	0	66 (2)
Language			
Arabic	152 (1)	<5^a^	28 (1)
English	21 318 (94)	1172 (99)	2306 (91)
Spanish	937 (4.1)	<5^a^	6 (1)
Other	201 (1)	<5^a^	38 (2)
Unknown, No.	<5^a^	0	234
Race			
American Indian or Alaska Native	66 (0.3)	6 (1)	<5^a^
Asian	331 (2)	5 (0.4)	36 (2)
Black or African American	5035 (22)	372 (31)	666 (27)
Hispanic, Latino, or Latina	1205 (5)	22 (2)	185 (8)
Middle Eastern or North African	179 (1)	<5^a^	22 (1)
White	15 343 (68)	747 (63)	1432 (59)
Multiracial	237 (1)	24 (2)	42 (2)
Other^b^	175 (1)	7 (1)	53 (2)
Unknown, No.	39	<5^a^	336
Ethnicity			
Hispanic, Latino, or Latina	1853 (8)	48 (4)	261 (13)
Not Hispanic, Latino, or Latina	20 352 (91)	1124 (95)	1736 (83)
Other^b^	219 (1)	8 (1)	89 (4)
Unknown, No.	186	5	690
Insurance			
Exchange	1098 (5)	94 (8)	93 (3)
Medicaid	3081 (14)	326 (28)	487 (18)
Medicare	6908 (31)	243 (21)	554 (20)
Pending Medicaid	143 (1)	30 (3)	57 (2)
Private	7591 (34)	71 (6)	685 (25)
Uninsured	2272 (10)	395 (33)	598 (22)
Worker’s Compensation	390 (2)	1 (0.08)	107 (4)
Other	1127 (5)	25 (2)	195 (7)
Arrival method			
Ambulance or hospital ground transportation	5745 (25)	424 (36)	707 (28)
Car	15 284 (68)	520 (44)	1398 (56)
Medical flight	582 (3)	8 (1)	213 (9)
Police	34 (0.2)	<5^a^	<5^a^
Public transportation	180 (1)	84 (7)	43 (2)
Taxi	80 (0.4)	<5^a^	11 (0.4)
Tiered or rapid response	168 (1)	5 (0.4)	26 (1)
Other	505 (2)	136 (11)	111 (4)
Unknown, No.	11	1	263
ED disposition			
Admission	9392 (43)	287 (25)	655 (28)
Deceased	6 (0.03)	0	39 (2)
Discharge	11 300 (51)	565 (48)	1009 (43)
External transfer	50 (0.2)	29 (3)	2 (0.07)
Internal transfer	28 (0.1)	0	177 (8)
Left after being seen	391 (2)	50 (4)	73 (3)
Left without being seen	441 (2)	39 (3)	378 (16)
Psychiatry transfer	463 (2)	198 (17)	35 (2)
Unknown, No.	539	17	408
Presenting symptom category			
Cardiac or pulmonary	4181 (19)	132 (11)	282 (12)
GI, GU, or kidney	4941 (22)	101 (9)	227 (10)
Infectious	1275 (6)	71 (6)	102 (4)
Intoxication	335 (2)	118 (10)	51 (2)
Metabolic or systemic	464 (2)	18 (2)	41 (2)
MSK or skin	2048 (9)	144 (12)	277 (12)
Neurologic	3140 (14)	81 (7)	319 (14)
Psychiatric	381 (2)	128 (11)	49 (2)
Social	13 (0.06)	27 (2)	12 (1)
Suicide	220 (1)	132 (11)	24 (1)
Trauma	3371 (15)	146 (12)	655 (28)
Other	2217 (10)	85 (7)	264 (11)
Unknown, No.	24	2	473
Primary ED diagnosis category			
Cardiac or pulmonary	3867 (18)	121 (11)	280 (14)
GI, GU, or kidney	4899 (22)	95 (8)	185 (9)
Infectious	2418 (11)	103 (9)	147 (7)
Intoxication	396 (2)	141 (12)	55 (3)
Metabolic or systemic	852 (4)	31 (3)	35 (2)
MSK or skin	1870 (9)	102 (9)	237 (12)
Neurologic	2787 (13)	82 (7)	310 (15)
Psychiatric	336 (2)	112 (10)	43 (2)
Social	24 (0.1)	38 (3)	25 (1)
Suicide	253 (1)	142 (12)	17 (1)
Trauma	3133 (14)	139 (12)	536 (27)
Other	1238 (6)	35 (3)	144 (7)
Unknown, No.	537	44	762
Triage score^c^			
1	278 (1)	7 (1)	391 (17)
2	5397 (24)	372 (31)	506 (22)
3	15 307 (68)	644 (54)	946 (42)
4	1575 (7)	141 (12)	367 (16)
5	49 (0.2)	21 (2)	52 (2)
Unknown, No.	4	0	514

Abbreviations: ED, emergency department; GI, gastrointestinal; GU, genitourinary; MSK, musculoskeletal.

^a^ Indicates fewer than 5 visits characterized within a cell to avoid inadvertently identifying participants.^32^

^b^ Included “none of these,” “unable to provide,” and “prefer not to answer.”

^c^ The triage score is presented as the Emergency Severity Index, with level 1 indicating most critically ill and level 5 indicating least ill.^33^ All data are presented at the level of the visit.

Visits with positive screening results were more likely to occur among patients who arrived at the hospital via ambulance or other hospital transportation (424 [36%] vs 5745 [25%]) than by car (520 [44%] vs 15 284 [68%]), with a notable percentage of visits among those arriving by public transportation (84 [7%] vs 180 [1%]) (Table 2).^32^ The ESI level was more likely to be 2 (372 [31%] vs 5397 [24%]) or 4 (141 [12%] vs 1575 [7%]). Visits with patients who screened positive were more likely to end with a transfer to psychiatric care (198 [17%] vs 463 [2%]). Chief concerns for a visit with a positive screening were more often suicide (132 [11%] vs 220 [1%]), another mental health concern (128 [11%] vs 381 [2%]), or intoxication (118 [10%] vs 335 [2%]). Similarly, the primary ED diagnosis was more likely to be related to suicide (142 [12%] vs 253 [1%]) or intoxication (141 [12%] vs 396 [2%]). Complete clinical characteristics are presented graphically in eFigure 2 in Supplement 1.

Visits with a missing housing screening (n = 2776) were more likely to be triaged as ESI level 1 than visits with positive screening results and those with negative screening results (391 [17%] vs 7 [1%] vs 278 [1%]) and to have a traumatic chief concern (655 [28%] vs 146 [12%] vs 3371 [15%]) or primary ED diagnosis (536 [27%] vs 139 [12%] vs 3133 [14%]) (Table 2).^32,33^ Visits with a missing housing screening were also more likely to end with the patient leaving without being seen (378 [16%] vs 39 [3%] vs 441 [2%]).

**Figure 1.**
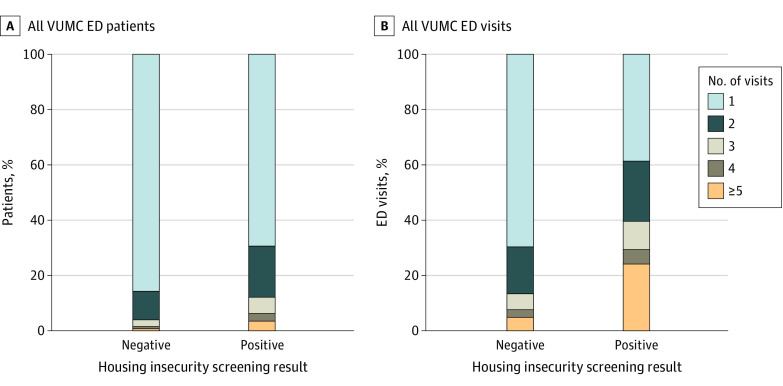
Utilization of the Vanderbilt University Medical Center (VUMC) Emergency Department (ED) Between January 5 and May 16, 2023 A and B, Three to 5 or more visits was considered high health care utilization. A, Percentage of individual patients who had 1 to 5 or more visits during the study period by housing insecurity and/or homelessness screening results at each visit. B, Percentage of VUMC ED visits that people with 1 to 5 or more visits used during the study period.

Of all 660 individual patients who screened positive for housing insecurity, 80 (11%) had 3 or more visits during the study period (high utilization) compared with 725 of 18 372 patients (4%) who screened negative (Figure 1A). Patients with high utilization accounted for 39% (469) of all visits with positive screening results compared with 13% (3027) of visits with negative screening results (Figure 1B). Among patients with a positive screening result, those with high utilization were more likely than those with a negative screening result to have pending (19 [4%] vs 42 [1%]) or active (145 [31%] vs 589 [19%]) Medicaid. This group was also more likely to present with a chief concern related to suicide (59 [13%] vs 49 [1.6%]).

**Figure 2.**
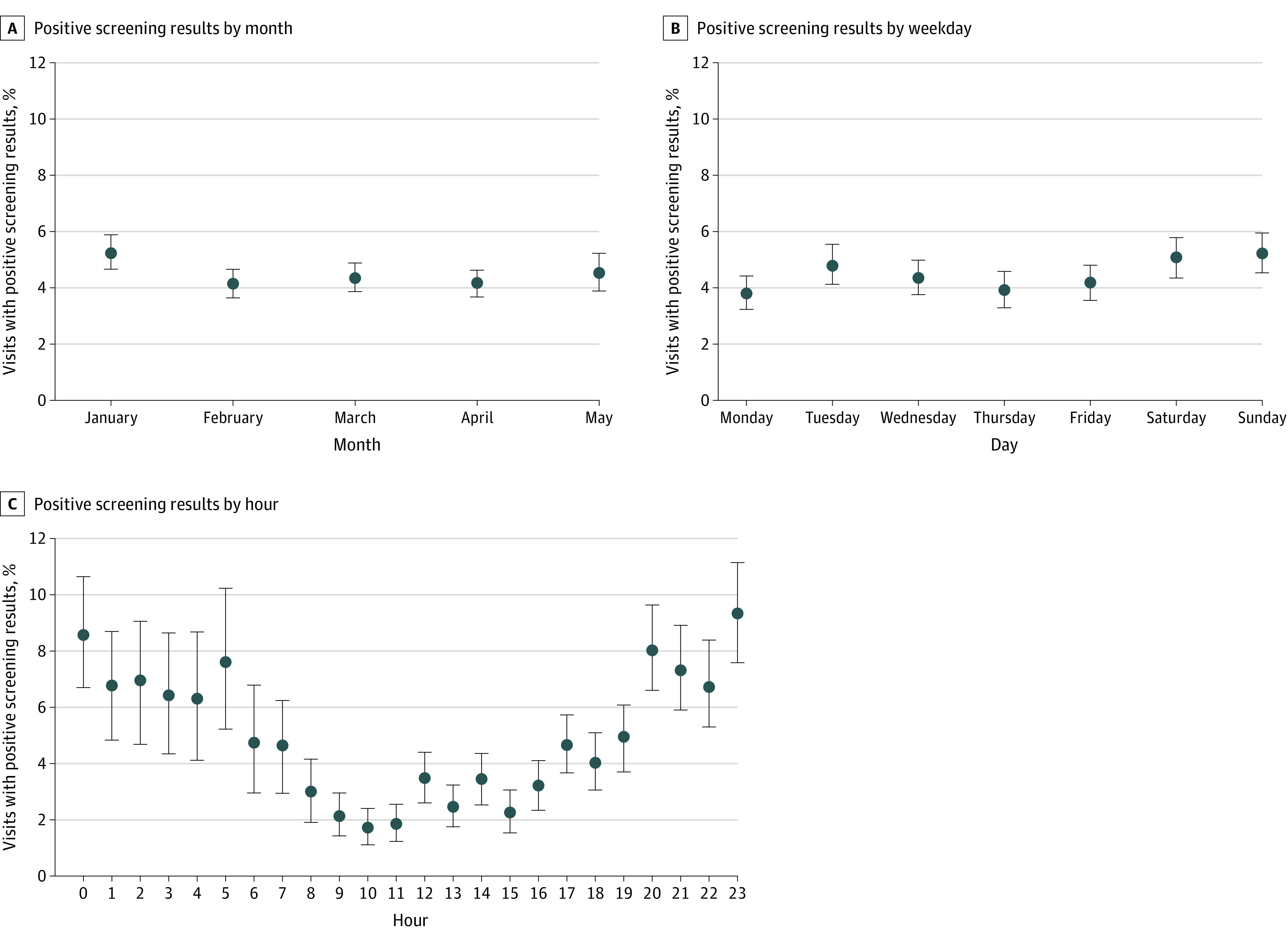
Visits With Patients Screening Positive for Housing Insecurity Between January 5 and May 16, 2023 Error bars indicate 1000 bootstrapped sample 95% CIs.

Although the data showed a relatively stable percentage of homelessness by month (Figure 2A) and day of the week (Figure 2B), a higher percentage of visits with positive screening results occurred from 8 pm to 6 am than from 6 am to 8 pm (Figure 2C). This finding could be due to the lower number of visits with negative screening results overall during those hours (eFigure 3 in Supplement 1). There was no meaningful association between the percentage of visits with a positive screening results and rainfall, minimum temperature, or maximum temperature (eFigure 4 in Supplement 1). Finally, the social work team was able to conduct evaluations at 919 of the 1185 visits (78%) among patients who screened positive for homelessness and/or housing insecurity.

## Discussion

To our knowledge, no universal method exists within EDs to identify patients experiencing housing insecurity and to systematically provide support for their needs. Screening for housing insecurity is uncommon among standard ED protocols, and housing status is rarely recorded in patient medical records.^19,34^ We believe that identifying housing instability, which often co-occurs with other structural inequalities,^35^ is the first step in delivering health care that truly addresses social determinants of health.

Although prior studies have found that patients experiencing homelessness are more likely to visit the ED than are patients who are housed,^15,25^ few have used data gathered from the EMR to understand this patient population.^6^ Through implementation of the 2-question HSCR within the ED at an urban Southeast US hospital, we found demographic and health care utilization differences between housed individuals and those experiencing housing insecurity.

### Homelessness and Housing Insecurity

Although the literature estimating the proportion of homelessness in health care settings is variable, our findings (5% positive) fall in the middle of the wide range of previously published rates.^22^ Most prior estimates of homelessness and housing insecurity were from oral surveys or indirect quantifications, such as “homeless” listed as the patient address or chief concern. Databases on randomly assigned 1-week reporting periods in which physicians collected patient data generated a lower estimate of 0.7% of all visits across the US.^22,36^ This discrepancy may stem from inconsistent physician querying of housing status or other sources of reporting bias. Conversely, 3 studies relying on randomly selecting individuals to interview in urban EDs found a higher proportion of people experiencing homelessness at 14% to 35%.^4,21,23^ This finding may indicate regional differences, variations between academic and community hospitals, or unconscious bias in who was approached to complete surveys. Conversely, our study relied on standardized questions during patient registration. In line with our findings, a large prospective study using components of the HSCR to screen consenting adults in 3 Pennsylvania EDs found that 7% were experiencing homelessness.^37^ Screening all ED patients using a protocolized, systematic strategy likely offers a more reliable quantification of patients experiencing homelessness than relying on diagnostic codes or inconsistent patient querying, a hypothesis that remains a key area of future work.

### Patient Characteristics

The higher proportion of male, middle-aged, and Black patients among those who screened positive is in line with previous studies and point-in-time counts in Nashville.^3,38^ As in previous studies,^38,39^ patients with positive screening results were also more likely to arrive by ambulance or public transportation. Transportation barriers faced by people experiencing homelessness may contribute to delayed access to care.^40,41^ Furthermore, use of ambulance transportation for nonemergency conditions makes emergency medical services vehicles and crews unavailable for patients who require urgent transport and may lead to increased health care costs and burnout among emergency medical services providers.^42^

Finally, as in the existing literature,^23^ positive screening results were more likely among uninsured patients or those enrolled in Medicaid. This finding may point to the continued need for Medicaid expansion and more widespread education regarding insurance eligibility for patients experiencing homelessness.

### Chief Concerns and Diagnoses

People experiencing homelessness are more likely than people who are housed to have mental illness, childhood trauma, decreased access to health care services, and substance use disorders, all of which are associated with suicide and substance use disorder risk.^43,44,45,46^ In line with other studies,^47,48,49^ ED visits for patients with housing insecurity or homelessness were more likely to include a chief concern and primary diagnosis related to suicide, intoxication, or trauma. Discharge was more likely to be to psychiatric care for those who screened positive than for those who screened negative. Finally, there was concordance between presenting chief concern and primary diagnosis, suggesting that homeless individuals are not at increased odds of malingering or fabricating symptoms to get food or shelter, as has been suggested by some prior studies.^46,50^ The proportion of visits with positive screening results at night suggests the continued need for support and homeless preparedness in EDs overnight.

Overall, these findings point to a need for more comprehensive psychiatric care, including suicide prevention and support for treating substance use disorders for patients experiencing homelessness as well as additional community support and bolstered coordination between EDs and mental health care facilities. Finally, continued efforts should be made to maintain resources overnight and bring mental health care directly to the street.^46^

### High Utilization

It is well established that patients experiencing homelessness access the ED more than patients who are housed and that patients categorized as having high utilization account for a sizable proportion of these visits.^15,24^ That was true in our study, in which people screening positive for homelessness were more likely to log 3 or more ED visits during the study period and represented a disproportionate proportion of visits.

This finding supports data suggesting that those experiencing homelessness are more likely to use the ED with greater frequency but, most importantly, points to a small proportion of individuals experiencing homelessness constituting a considerable proportion of visits. If additional resources can be directed to this relatively small group of individuals, the impact could be significant. That patients with high utilization were more likely to present with a primary diagnosis related to suicide and to be insured with Medicaid further supports the need for improved mental health care and coordination with psychiatric hospitals and insurers.

Future studies should aim to include more institutions and regions. One evaluation of 2 screening tools across 11 sites found that some patients screened positive for homelessness with one tool but not the other and advised caution when selecting a screening tool, as homelessness does not have a single, universal definition.^51^ Accordingly, in addition to providing a more accurate picture of homelessness and housing insecurity in the region, we hope this study may serve as a call for the implementation of standardized screening for homelessness across all EDs. Understanding details of the previously underrecognized population experiencing homelessness will enable our center to continue directing resources to improve health and social conditions that lead to health inequities. Specifically, we are using these data to develop a multidisciplinary homeless health care team that includes in-hospital and street medical care for people experiencing homelessness and housing insecurity. Findings from this study point to the importance of homeless health and street medicine initiatives within hospitals, especially to target patients who use the ED regularly. They highlight the need for robust coordination between EDs and psychiatric hospitals and continued advocacy for targeted resources for these patients. Through systematic screening and connection to social work, we hope to advance health equity, reduce health care costs, and improve health outcomes for patients experiencing homelessness.

### Strengths and Limitations

Our study has strengths, including the implementation of our screening process (leading to 90% completion). Through interviews, we found that registration staff felt that the screening fit easily into their workflow, social workers were motivated because they were helping more patients experiencing homelessness, and while many physicians did not seem aware of the screening, they did alter their care for patients who they identified as experiencing homelessness.

This study has several limitations. First, housing screenings were marked as “unable to obtain” at 10% of visits, likely due to these patients arriving with immediate, life-threatening illness or trauma or leaving without formal discharge. An ongoing quality improvement project aims to better understand patients with missing screenings. Second, as a highly specialized referral center,^26^ VUMC is frequently full (diverting ambulances to other local or regional hospitals), which may underestimate the prevalence of ED visits by people experiencing homelessness in the region. Third, data such as primary diagnosis and chief concern of the patient were obtained retrospectively from the EMR and are therefore prone to misclassification. Notably, as a descriptive study, we are not able to imply causation but rather associations between primary diagnosis, chief concern, demographic information, or weather and housing status.

## Conclusions

In this cross-sectional study, through implementation of a universal screening process in the ED using registration and the EMR, we identified and described the population with housing insecurity presenting to the ED. Of 23 795 ED visits, at 5%, patients screened positive for housing insecurity and were more likely to present with a chief concern of suicide, to be uninsured, and to have multiple visits during the study period.

## Notes

Supplement 1. **eFigure 1.** Demographic Characteristics of Patients at VUMC Emergency Department Visits Between January 5 and May 16, 2023 **eFigure 2.** Clinical Characteristics of Patients at VUMC Emergency Department Visits Between January 5 and May 16, 2023 **eFigure 3.** Absolute Counts and Percentages of Visits by Month, Day, and Hour Between January 5 and May 16, 2023 **eFigure 4.** Proportion of Positive Screens by Rainfall, Minimum Temperature, and Maximum Temperature

Supplement 2.
Data Sharing Statement
