# Supplementary material for: Characteristics and Health Care Utilization of Patients With Housing Insecurity in the ED
Source: JAMA Netw Open. 2024 Apr 26;7(4):e248565. doi: 10.1001/jamanetworkopen.2024.8565 (PMC11053378; doi:10.1001/jamanetworkopen.2024.8565)
Supplement: Supplement 1. — eFigure 1. Demographic Characteristics of Patients at VUMC Emergency Department Visits Between January 5 and May 16, 2023 eFigure 2. Clinical Characteristics of Patients at VUMC Emergency Department Visits Between January 5 and May 16, 2023 eFigure 3. Absolute Counts and Percentages of Visits by Month, Day, and Hour Between January 5 and May 16, 2023 eFigure 4. Proportion of Positive Screens by Rainfall, Minimum Temperature, and Maximum Temperature [file jamanetwopen-e248565-s001.pdf]

# Supplementary Online Content

Ball MAZ, Sack DE, Druffner SA, et al. Characteristics and health care utilization of patients with housing insecurity in the ED. *JAMA Netw Open.* 2024;7(4):e248565. doi:10.1001/jamanetworkopen.2024.8565

**eFigure 1.** Demographic Characteristics of Patients at VUMC Emergency Department Visits Between January 5 and May 16, 2023
**eFigure 2.** Clinical Characteristics of Patients at VUMC Emergency Department Visits Between January 5 and May 16, 2023
**eFigure 3.** Absolute Counts and Percentages of Visits by Month, Day, and Hour Between January 5 and May 16, 2023
**eFigure 4.** Proportion of Positive Screens by Rainfall, Minimum Temperature, and Maximum Temperature

This supplementary material has been provided by the authors to give readers additional information about their work.

© 2024 Ball MAZ et al. *JAMA Network Open.*

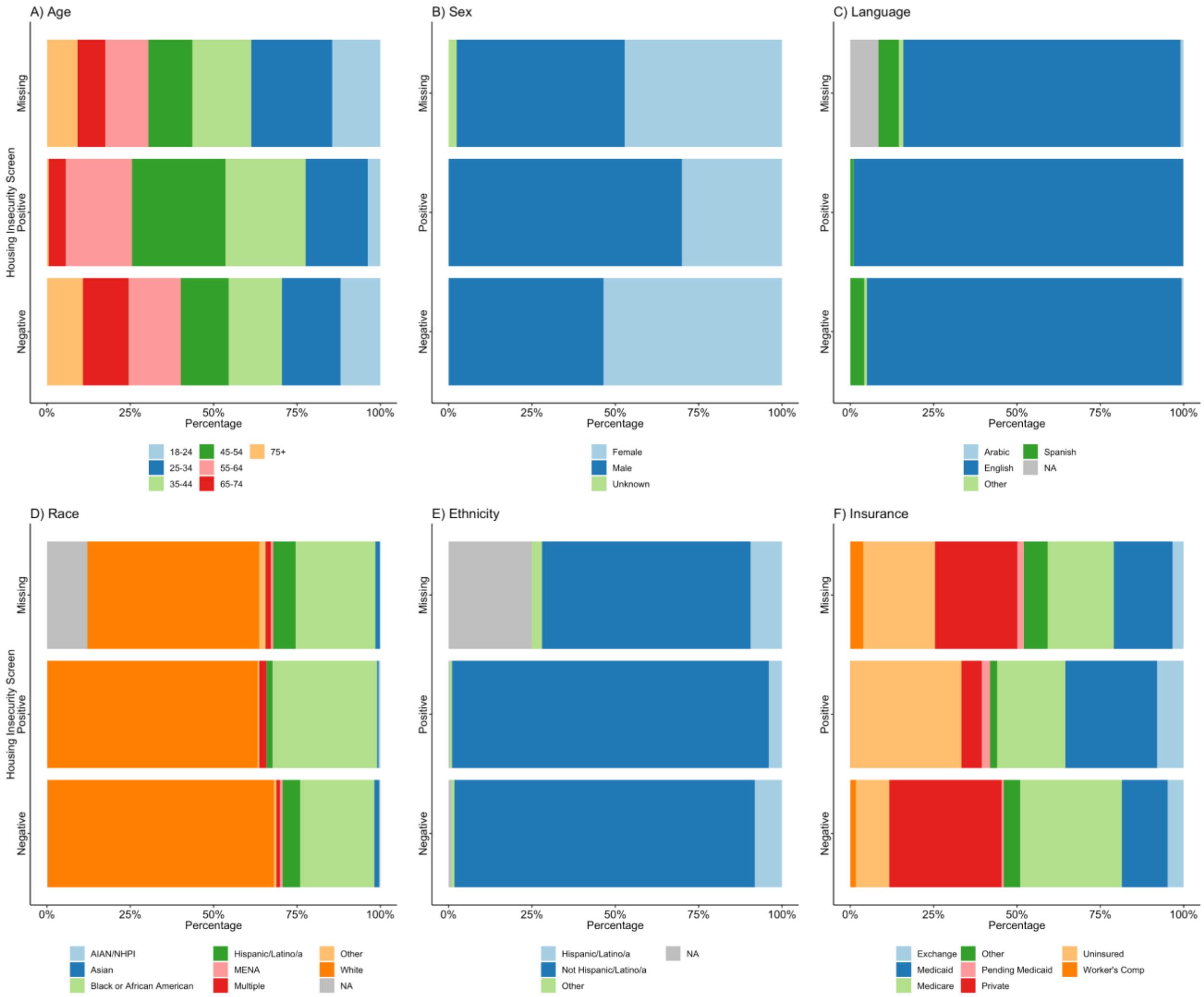


**eFigure 1.** Demographic Characteristics of Patients at VUMC Emergency Department Visits Between January 5 and May 16, 2023. Panel A shows the ages of patients presenting by month and housing insecurity and/or homelessness status during the study period. Panel B shows their sex, Panel C their language, Panel D their race, Panel E their ethnicity, and Panel F their insurance as recorded in the electronic medical record at that visit. On panel E, “Hispanic/Latino/a” includes patients who described their ethnicity as “Cuban”, “Hispanic or Latino”, “Mexican”, “Puerto Rican” and “Other Hispanic, Latino/a, or Spanish origin.” Abbreviations: AIAN – American Indian or Alaska Native; NHPI -- Native Hawaiian or Other Pacific Islander; MENA – Middle Eastern or North African.

© 2024 Ball MAZ et al. *JAMA Network Open.*

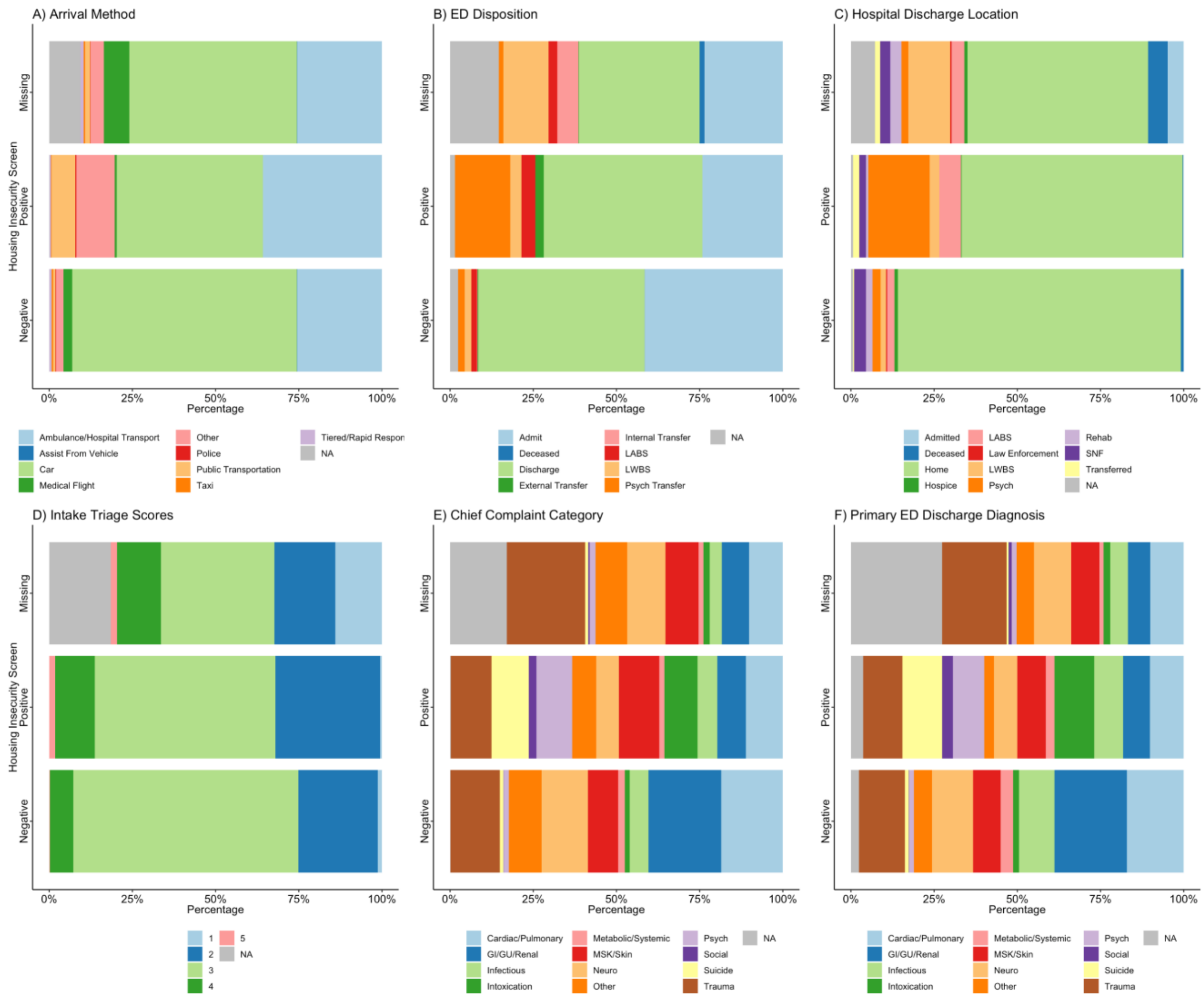


**eFigure 2.** Clinical Characteristics of Patients at VUMC Emergency Department Visits Between January 5 and May 16, 2023. Panel A shows how patients arrived for each VUMC emergency department (ED) visit during the study period by housing insecurity and/or homelessness status. Panel B shows their ED disposition, Panel C their hospital disposition, Panel D their intake Emergency Severity Index (ESI) triage score with level I being the most critically ill, Panel E their Chief Complaint, and Panel F their primary ED diagnosis at discharge as recorded in the electronic medical record at that visit. Abbreviations: LABS – Left after being seen; LWBS – Left without being seen; SNF – Skilled nursing facility; GI – Gastrointestinal; GU – Genitourinary; MSK – Musculoskeletal

© 2024 Ball MAZ et al. *JAMA Network Open.*

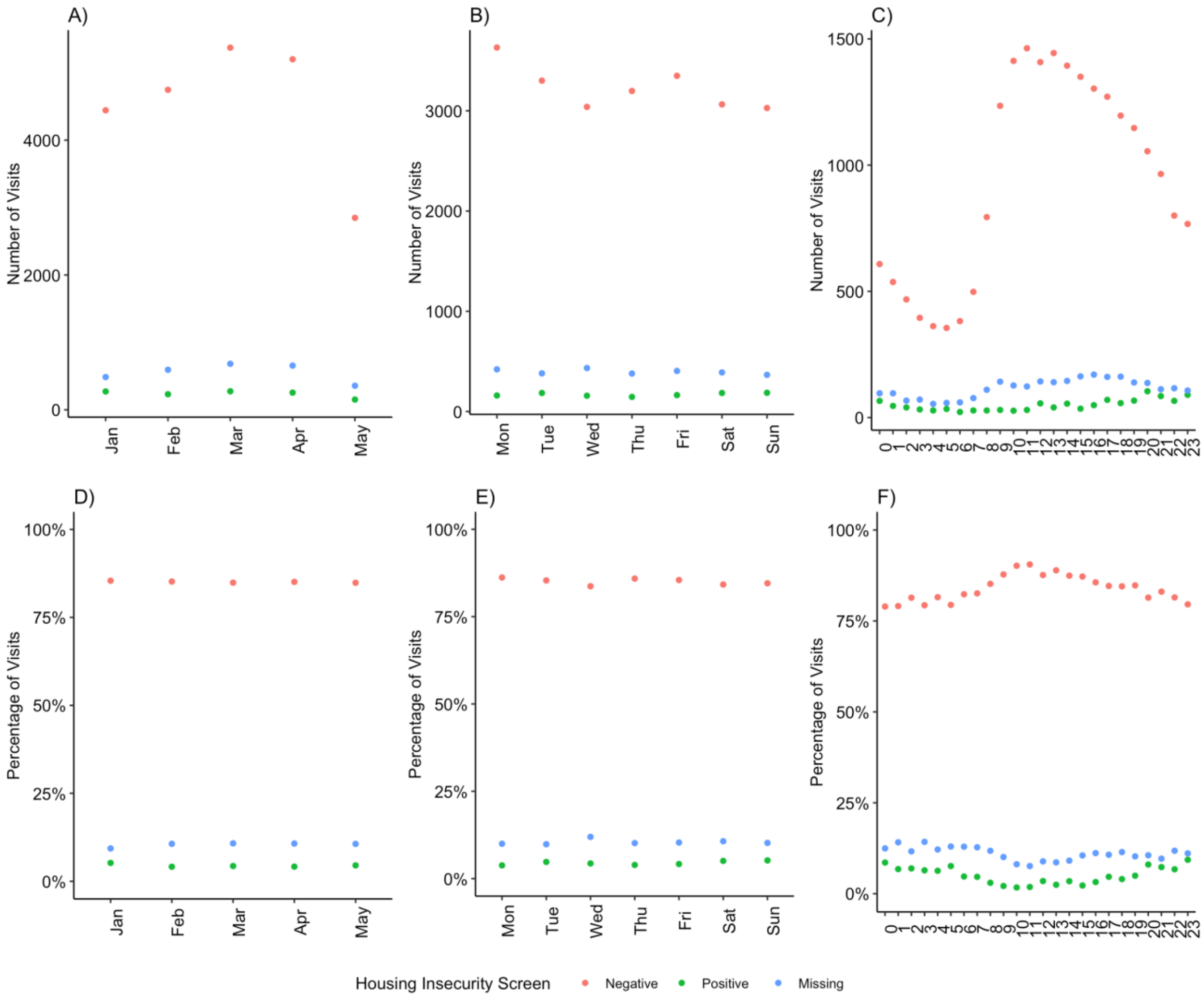


**eFigure 3.** Absolute Counts and Percentages of Visits by Month, Day, and Hour Between January 5 and May 16, 2023

Panels A and D show the raw number (A) and percentage (D) of visits among those who screened negative (red), positive (green), and had a missing screen (blue) for housing insecurity by month. Panels B (raw number) and E (percentage) show the same data by day of the week and Panels C (raw number) and F (percentage) do so by hour of the day.

© 2024 Ball MAZ et al. *JAMA Network Open.*

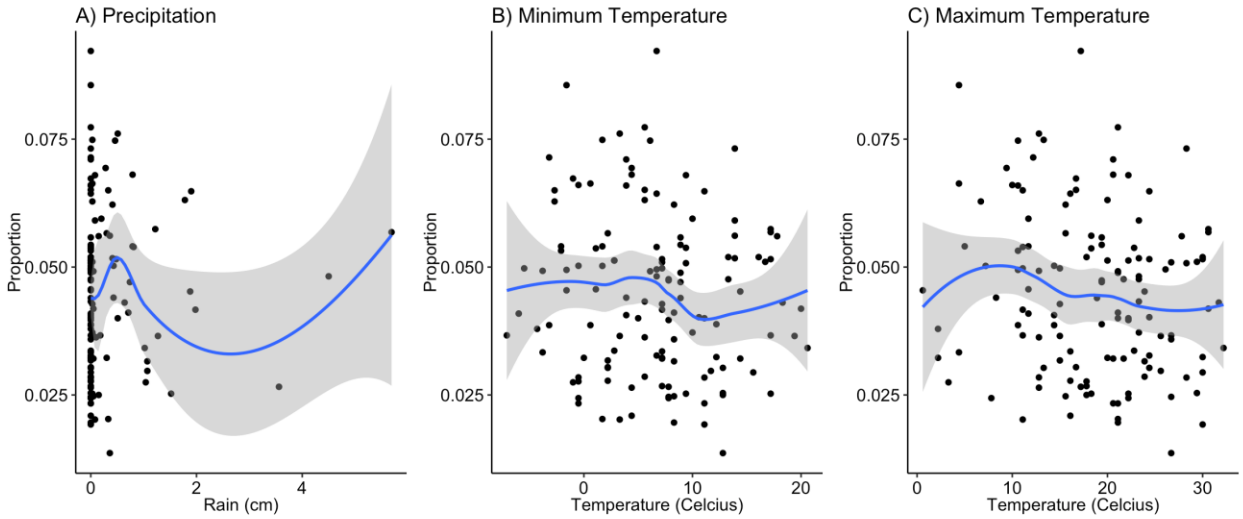


**eFigure 4.** Proportion of Positive Screens by Rainfall, Minimum Temperature, and Maximum Temperature

Panel A shows proportion of positive screens by rainfall in centimeters, Panels B and C show proportion of positive screens by low and high temperatures. Rainfall and temperature data was acquired from the National Oceanic and Atmospheric Administration at the Nashville International Airport weather station. The blue line represents the locally estimated scatterplot smoothing (loess) line of best fit and the shaded area represent the associated 95% confidence interval.

© 2024 Ball MAZ et al. *JAMA Network Open.*
