# Supplementary material for: Characteristics and Health Care Utilization of Patients With Housing Insecurity in the ED
Source: JAMA Netw Open. 2024 Apr 26;7(4):e248565. doi: 10.1001/jamanetworkopen.2024.8565 (PMC11053378; doi:10.1001/jamanetworkopen.2024.8565)
Supplement: Supplement 2. — Data Sharing Statement [file jamanetwopen-e248565-s002.pdf]

Data Sharing Statement

Ball. Characteristics and Health Care Utilization of Patients With Housing Insecurity in the ED. *JAMA Netw Open*. Published April 26, 2024. doi:10.1001/jamanetworkopen.2024.8565

Data
Data available: No

Additional Information
Explanation for why data not available: Given the potential for patient identification, the data will not be shared at this time. Should editors wish to revisit this decision, we would gladly discuss a strategy to share data without the risk of releasing sensitive PHI. Statistical analysis is open and available and can be viewed at: https://github.com/dannysack/ed_housing_screen.
